# Efficient Activation of Apoptotic Signaling during Mitotic Arrest with AK301

**DOI:** 10.1371/journal.pone.0153818

**Published:** 2016-04-20

**Authors:** Avijeet Chopra, Michael J. Bond, Marina Bleiler, Michelle Yeagley, Dennis Wright, Charles Giardina

**Affiliations:** 1 Department of Molecular and Cell Biology, University of Connecticut, 91 N Eagleville Rd, Storrs, Connecticut, United States of America; 2 School of Pharmacy, University of Connecticut, 69 N Eagleville Rd, Storrs, Connecticut, United States of America; Winship Cancer Institute of Emory University, UNITED STATES

## Abstract

Mitotic inhibitors are widely utilized chemotherapeutic agents that take advantage of mitotic defects in cancer cells. We have identified a novel class of piperazine-based mitotic inhibitors, of which AK301 is the most potent derivative identified to date (EC_50_ < 200 nM). Colon cancer cells arrested in mitosis with AK301 readily underwent a p53-dependent apoptosis following compound withdrawal and arrest release. This apoptotic response was significantly higher for AK301 than for other mitotic inhibitors tested (colchicine, vincristine, and BI 2536). AK301-treated cells exhibited a robust mitosis-associated DNA damage response, including ATM activation, γH2AX phosphorylation and p53 stabilization. The association between mitotic signaling and the DNA damage response was supported by the finding that Aurora B inhibition reduced the level of γH2AX staining. Confocal imaging of AK301-treated cells revealed multiple γ-tubulin microtubule organizing centers attached to microtubules, but with limited centrosome migration, raising the possibility that aberrant microtubule pulling may underlie DNA breakage. AK301 selectively targeted *APC*-mutant colonocytes and promoted TNF-induced apoptosis in p53-mutant colon cancer cells. Our findings indicate that AK301 induces a mitotic arrest state with a highly active DNA damage response. Together with a reversible arrest state, AK301 is a potent promoter of a mitosis-to-apoptosis transition that can target cancer cells with mitotic defects.

## Introduction

Mitosis is an intricate process in actively dividing cells, orchestrating a myriad of kinases and signaling pathways. Ascribing to this complexity, mitosis is a particularly sensitive phase of the cell cycle [1]. A number of mitotic checkpoints ensure the fidelity of chromosome segregation and cytokinesis; failure of mitotic checkpoints often results in chromosomal alterations, culminating in mitotic catastrophe or cancer-promoting chromosomal instability [2, 3]. Cancer cells often lack important cell cycle checkpoints and may execute mitosis with improper spindle assembly [2]. Therefore, mitotic inhibitors are among the most widely utilized chemotherapeutic agents in the treatment of a number of malignancies [1]. Despite their widespread use, the activity of present mitotic inhibitors is limited by their low activity and associated toxicity. The response of the cancer cells to mitotic inhibitors can be distinctly different with varying magnitudes of effect–some cells remain arrested in mitotic phase, while others exit division and undergo apoptosis [4, 5]. How microtubule disrupting agents result in apoptosis and what cellular factors influence the transition of mitotic arrest to apoptosis is not completely understood. Previous studies indicate that activation of the spindle assembly checkpoint (SAC) during mitosis is necessary for an efficient induction of apoptosis, often through the activation of the tumor suppressor protein p53 [6, 7]. It is important to elucidate the signaling pathways that associate aberrant mitosis and apoptosis, and how these pathways are affected by mitotic defects in cancer cells. This information may reveal opportunities that could be exploited for the development of novel therapeutics that target aberrant mitotic regulation in cancer cells.

While screening for molecules that might be able to accentuate colon cancer cell sensitivity to the inflammatory microenvironment of a cancer, we identified a family of small molecule inhibitors that dramatically enhanced colon cancer cell death in the presence of TNF and related death ligands [8, 9]. The most potent of these compounds, AK301 had activity in the nanomolar range (EC_50_ ≈ 115 nM) [8]. Notably, AK301 was found to arrest colon cancer cells in a mitotic state that was acutely sensitive to TNF. Further investigation of AK301 showed a robust activation of apoptosis in a p53-normal colon cancer cell line (HCT116 cells), simply by removing AK301 from the medium and releasing the cells from mitotic arrest. Apoptosis induced by this mitotic arrest-and-release protocol was significantly greater than that induced by other mitotic inhibitors tested. Here we characterize the arrest state induced by AK301 to determine the basis of its relationship with apoptosis. We report that colon cancer cells treated with AK301 arrest at a mitotic state with high levels of ATM activation and p53 stabilization. The stabilization of p53 during mitosis culminates in an apoptotic response in cells following AK301 withdrawal, which releases cells from mitosis given the readily reversible arrest state induced by AK301. We propose that AK301 and its derivatives will be beneficial for probing how apoptotic signaling via the ATM-p53 pathway can be activated during mitosis. A better understanding of this pathway may ultimately be exploitable for developing novel therapies aimed at treating cancers with mitotic defects.

## Materials and Methods

### Cell Culture

HT29 and HCT116 colon cancer cell lines were obtained from the American Type Culture Collection (Manassas, VA). HT29 and HCT116 cell lines were cultured in McCoy’s 5A medium, with 10% fetal bovine serum, non-essential amino acids and antibiotic/antimycotic (Life Technologies, Guilford, CT). Immortalized primary colon cell lines Young Adult Mouse Colonocytes (YAMC) and Immorto-Mouse Colonic Epithelial Cells (IMCE) were a gift from Dr. R Whitehead (Vanderbilt University, Nashville, TN)[10, 11]. YAMC and IMCE cells were cultured in RPMI medium containing 5% fetal bovine serum, non-essential amino acids, antibiotic/antimycotic, insulin-transferrin-selenium (Life Technologies), and 5 units of murine gamma interferons. The cells were grown at 33°C. AK301 was synthesized from compounds obtained from the ChemBridge DIVERSet^TM^ library (San Diego, CA). Colchicine, Vincristine, and BI 2536 were obtained from Sigma Aldrich (St. Louis, MO), Acros Organics (Pittsburgh, PA), and SelleckChem Chemicals (Houston, TX), respectively. Drug treatments were performed approximately 24 h after passage for 16 h, unless otherwise indicated. TNF was obtained from Pierce Protein Research Products (Rockford, IL).

### Immunofluorescence microscopy

Cells cultured on coverslips were fixed with 4% paraformaldehyde at room temperature or 100% ice cold methanol at 4°C and then permeabilized with 0.5% Triton X-100 in PBS. Cells were blocked in 5% serum (in PBS) and then incubated with primary antibody (in 5% serum) on shaker for 1 h at room temperature. The following antibodies were used for these studies: phospho-histone H3 Ser 28 (sc-12927, Santa Cruz Biotechnology, Santa Cruz, CA), β-tubulin (E7 monoclonal antibody, Developmental Studies Hybridoma Bank, Iowa), γH2AX (sc-101696, Santa Cruz Biotechnlogy), γ-tubulin (GTU-88, Abcam, Cambridge, Massachusetts), Aurora B (ab2254, Abcam). Aurora A (630938, BD Transduction Laboratories). Appropriate secondary antibodies (Molecular Probes, Life Technologies or Jackson ImmunoResearch, West Grove, PA) were used for 45 min incubation. Nuclei were visualized using DAPI (5 μg/ml in PBS; DI306, Life Technologies). Coverslips were mounted on slides using ProLong Gold Antifade Reagent (Life Technologies). Images were acquired using Nikon A1R Confocal Microscope (version 2.11, Nikon Instruments Inc.) and NIS-Elements Advanced Research Software (version 4.13.01, build 916, Nikon Instruments Inc.). Quantification of immunostaining was performed using ImageJ image analysis software (http://rsb.info.nih.gov/ij) as previously described [12]. Following background subtraction and image stacking, both DAPI and immunofluorescence images were merged. Image brightness and contrast were modified with Adobe Photoshop software CC 2014 (Adobe Systems).

### Flow cytometry and cell cycle analysis

Cells were stained for γH2AX using the protocol described above for immunofluorescence staining. Briefly, cells were fixed with 4% paraformaldehyde, permeabilized with 0.1% Triton X-100, and blocked with 5% donkey serum. Cells were then incubated with γH2AX antibody (sc-101696, Santa Cruz Biotechnology) followed by incubation with Alexa Fluor^®^ 488 secondary antibody (Life Technologies). Cells were then harvested using trypsin-EDTA for 15 min at 37°C and washed once with PBS. Propidium iodide (30 μg/ml) was added to the cells prior to filtration through 35 μm cell strainer tubes. Cell were promptly analyzed by flow cytometry.

For cell cycle analyses, cells were analyzed for DNA content by ethanol fixation and staining with propidium iodide as previously described [8]. Cells were harvested using trypsin-EDTA, centrifuged at 1000 *X g* for 10 min and resuspended in 500 μl of cold saline GM. Cells were washed once with 1X PBS and then fixed for at least 2 hrs at -20°C in 3X volumes of cold 100% ethanol while vortexing. Cells were then pelleted and washed once with PBS containing 5 mM EDTA. Pelleted cells were stained with 30 μg/ml propidium iodide (Molecular Probes, Life Technologies Corp.) and 0.3 mg/ml RNase A (Sigma-Aldrich, St. Louis, MO) in 500 μl PBS solution for 40 min in the dark at RT. The stained cells were filtered through 35 μm cell strainer tubes (BD Biosciences, San Jose, CA). All flow cytometric analyses were performed on FACSCalibur (BD Biosciences) using Cell Quest software (BD Biosciences). The data were analyzed using FlowJo (v10, TreeStar Inc., Ashland, OR).

### Caspase-3 assay

Caspase-3 activity was determined as previously described [9]. Cells were collected, centrifuged at full speed, and washed once with PBS. Pelleted cells were lysed by two rounds of freeze-thaw in lysis buffer containing 10 mM Tris-HCl (pH 7.5), 0.1 M NaCl, 1 mM EDTA, and 0.01% Triton X-100 and centrifuged at 10,000 *X g* for 5 min. The assays were performed on 96 well plate by mixing 50 μl of lysis supernatant with 50 μl of 2X reaction mix (10 mM PIPES pH 7.4, 2 mM EDTA, 0.1% CHAPS, 10 mM DTT) containing 200 nM of the fluorogenic substrate Acetyl-Asp-Glu-Val-Asp-7-Amino-4-methylcoumarin (DEVD-AMC; Enzo Life Sciences). The fluorescence was quantified at the start of the reaction and after 30 min. Protein concentrations were determined using CBQCA Protein Quantitation Kit (Life Technologies). Caspase activity was determined by dividing the change in fluorescence by total protein content of the reaction mixture.

### Western blot

RIPA buffer was used for total protein extraction. 20 μg of protein was denatured under reducing conditions and separated on 10% polyacrylamide gels (Bio-Rad Laboratories, Hercules, CA) and transferred to nitrocellulose by voltage gradient transfer. The resulting blots were blocked with 5% (w/v) non-fat dry milk in PBS + 0.1% (v/v) Tween-20. Specific proteins were detected with appropriate antibodies using SignalFire^TM^ Elite ECL Reagent (Cell Signaling Technology). Immunoblotting antibodies were p53 (OP03, Calbiochem, Massachusetts), p-p53 (9284, Cell Signaling Technology, Massachusetts), ATM (2873, Cell Signaling Technology), and p-ATM Ser1981 (13050, Cell Signaling Technology), p21 (C-19, Santa Cruz Biotechnology, California), Bax (P-19, Santa Cruz Biotechnology), Bak (G-23, Santa Cruz Biotechnology), Mdm2 (OP115, Calbiochem), β-actin (I-19, Santa Cruz Biotechnology).

### Statistical analyses

One-way analysis of variance (ANOVA) was used when comparing two groups with Tukey’s post hoc test. For more than two groups, two-way ANOVA was used with Bonferroni correction for multiple comparisons. Significance was calculated at an alpha of 0.05.

## Results

### AK301-arrested cells show increased caspase-3 activity

**Fig 1:**
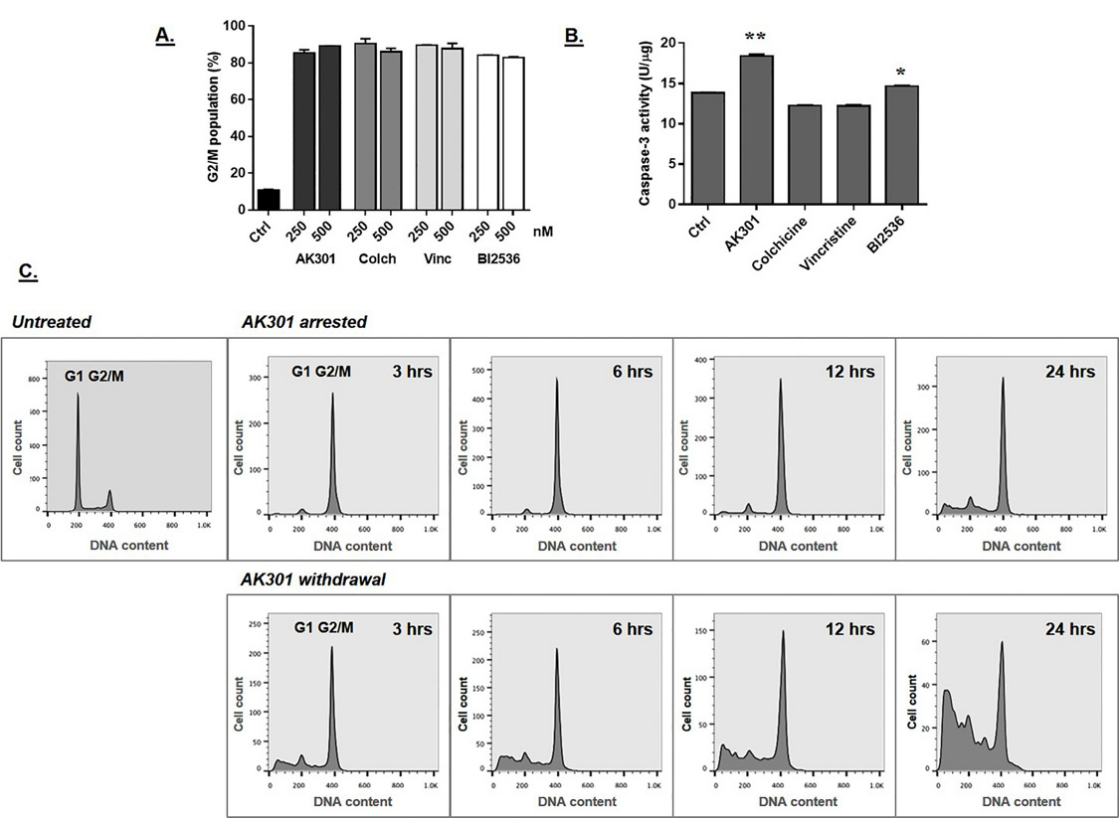
**A)** G2/M arrest in HCT116 colon cancer cells. HCT116 cells were treated with the indicated concentrations of AK301, colchicine or vincristine (microtubule inhibitors), or BI2536 (a PLK1 inhibitor) for 16 hours. Cells were then fixed and stained with propidium iodide (PI), and analyzed by flow cytometry. All four drugs induced high levels of G2/M arrest at both concentrations (P < 0.0001) with no significant differences between the compounds. **B)** HCT116 cells were treated with 500 nM of each of the indicated compounds for 16 h. Cell lysates were prepared and tested for caspase-3 activity using DEVD-AMC fluorogenic assay. AK301 (**P < 0.0001) and BI2536 (*P < 0.05) induced significantly higher levels of caspase-3 activation relative to control cells. **C)** Apoptosis in AK301 treated cells released from arrest. The left-most panel shows the cell cycle distribution of HCT116 cells under normal growth conditions. In the remaining panels, HCT116 cells were treated with 500 nM of AK301 for 16 hours. AK301 was then removed and cells were allowed to grow in fresh medium for 3, 6, 12, and 24 hours (bottom panel) or returned to AK301-containing medium (top panel). Cells were harvested at the indicated times following the medium change. Cells maintained in AK301 show a relatively stable G2/M arrest, whereas those switched to new medium showed increasing levels of sub-G1 apoptotic cells.

We were interested in determining how AK301 compared to other mitotic arrest agents with regard to its ability to activate apoptotic signaling. We therefore tested a collection of antimitotic agents, including microtubule inhibitors (colchicine and vincristine), and a PLK1 inhibitor (BI2536)[13]. Previous work in our lab showed that these compounds could all induce maximal G2/M arrest at concentrations of 250 nM and higher [9, 14]. As shown in Fig 1A, flow cytometric analysis of HCT116 treated with either 250 nM or 500 nM of these agents induced a G2/M arrest in over 80% of the cells (P < 0.0001). To examine the relationship between induced mitotic arrest and apoptotic signaling, we tested these agents for their ability to induce capase-3 activation using a DEVD-AMC fluorogenic substrate at 500 nM. As shown in Fig 1B, of the four mitosis-arresting agents, AK301 induced the highest levels of caspase-3 activity (P < 0.0001). Capsase-3 activity suggested a higher apoptotic potential of AK301 relative to the other arrest agents.

### AK301 withdrawal enhances apoptotic response in HCT116 cells

**Fig 2:**
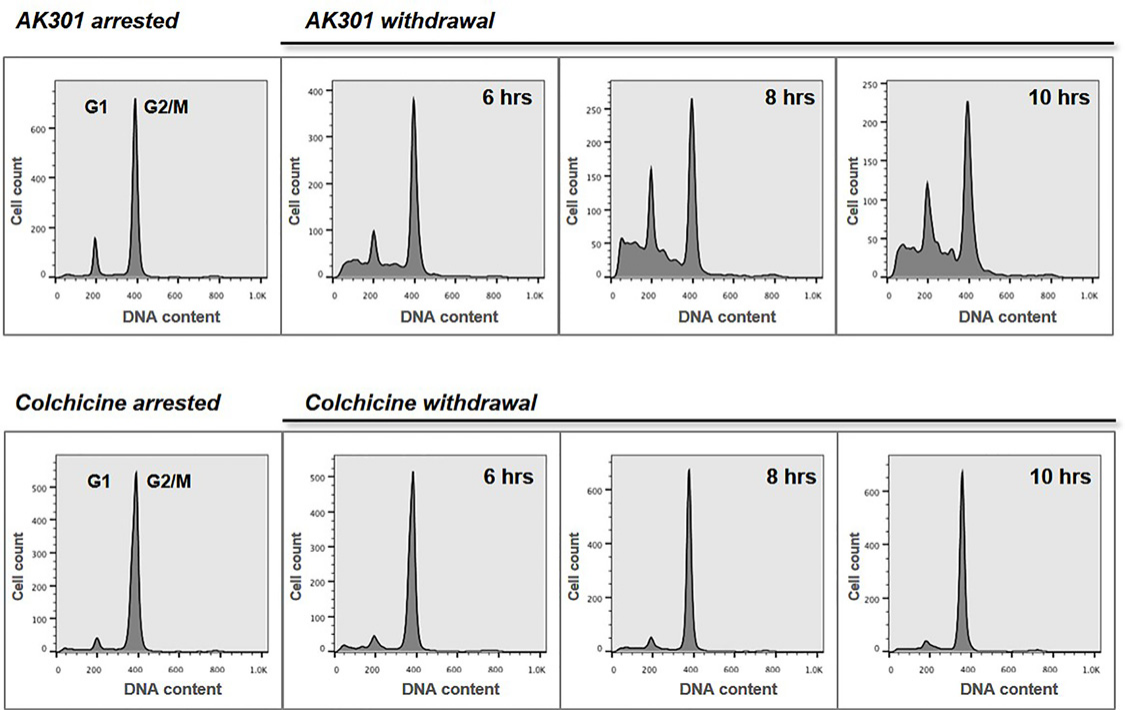
AK301 withdrawal induces more apoptosis than colchicine withdrawal. HCT116 cells were treated with 500 nM of AK301 or colchicine for 16 hours, as indicated. Cells were then switched to fresh growth medium for the indicated lengths of time. Flow cytometric analysis of DNA content showed that both AK301 and colchicine arrested HCT116 cells in G2/M phase of the cell cycle. However, upon drug withdrawal, cells arrested with AK301 showed the formation of more sub-diploid cells than those released from colchicine arrest.

**Fig 3:**
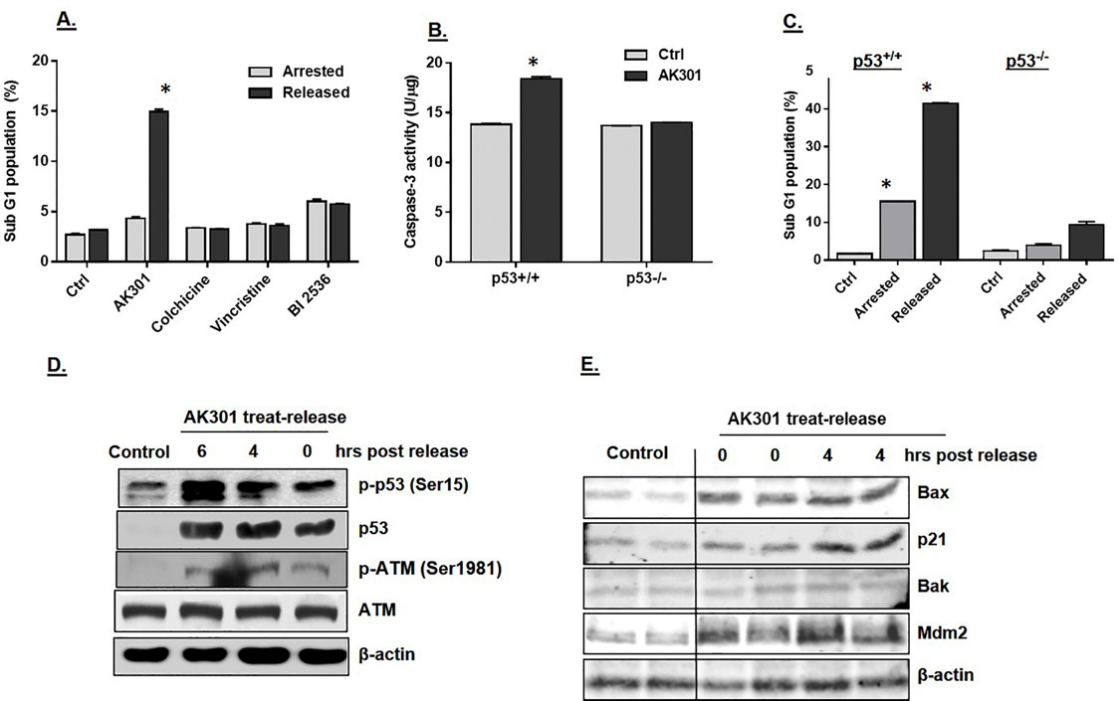
**A)** Apoptosis of cells exiting mitotic arrest. HCT116 cells treated with 500 nM of each of the indicated compounds for 16 hours. Cells where then either switched to drug-free medium for 8 hours, or treated with fresh drug-containing media. Flow cytometeric analysis of the DNA content showed that AK301 treated cells showed a significantly higher levels of apoptosis after release than cells treated with the other compounds (*P < 0.0001). **B)** Wild type and p53-null HCT116 cells were treated with 500 nM of AK301 for 16 hours. Cell lysates were prepared and tested for caspase-3 activity using DEVD-AMC fluorogenic assay. The p53-normal HCT116 cells showed more caspase-3 activation than the null cells (*P < 0.001). **C)** Wild type and p53-null HCT116 cells were treated and released with AK301 as described in 3A. Cells were then processed for flow cytometric analysis. Apoptosis was significantly higher in p53-normal HCT116 cells (*P<0.001). **D)** ATM activation and p53 stabilization following AK301 treatment. HCT116 cells were treated with 500 nM AK301 for 16 hours, followed by transfer into fresh medium for 0, 4, or 6 hours. Protein was then extracted for analysis. Immunoblot analysis shows phosphorylation of ATM at Ser1981 and phosphorylation and stabilization of p53 (p-p53 Ser15) in treated and released cells. β-actin was used as a loading control. **E)** Activation of p53 target genes by AK301. HCT116 cells (in biological duplicates) were treated with AK301 as in 3D, with and without a 4 hour release. Expression of Bax, Bak, p21 and Mdm2 was then determined by western blotting. β-actin was used as a loading control.

Since cells may not undergo apoptosis while arrested in mitosis, we withdrew AK301 from arrested HCT116 cells in culture and monitored their progression through the cell cycle. Flow cytometric analysis showed that ~85% of cells were in G1 prior to treatment, with very little sub-G1 cells (Fig 1C, left-most panel). However, at 3, 6, 12, and 24 hours post AK301 withdrawal, sub-G1 cells appeared as early as 3 hours, which progressed through 24 hours (Fig 1C, bottom panels). Cells maintained in AK301 showed persistent arrest with low levels of apoptosis during the 24 hour period (Fig 1C, top panels). We compared the apoptotic effect of AK301 to that of colchicine. HCT116 cells were treated with AK301 or colchicine to induce arrest, and were then analyzed post compound withdrawal. As shown in Fig 2, cells subjected to the AK301-arrest and release protocol showed a larger apoptotic sub-G1 population than cells released from a colchicine treatment, which remained arrested in G2/M. In addition, some AK301-treated cells underwent cell division (increased proportion of G1 cells from 3 to 24 hours), indicating that mitotic arrest by AK301 is more reversible than arrest induced by colchicine. Fig 3A compares the apoptotic-inducing ability of AK301 to the mitotic inhibitors colchicine, vincristine, and BI2536 in the arrest-and-withdrawal procedure. AK301 was found to be significantly more potent than these other agents.

### The role of p53 in AK301-induced apoptosis

To determine whether AK301-induced apoptosis was p53-dependent, we compared the effect of AK301 on caspase-3 activation in p53-normal and mutant cells. As shown in Fig 3B, AK301 caused an increase in capsase-3 activation in p53-normal HCT116 cells, but not in p53-null HCT116 cells. Likewise, in the AK301 treatment-and-release procedure, p53-normal HCT116 cells underwent a higher level of apoptosis than their p53-mutant counterparts (Fig 3C). Finally, western blot analysis showed that p53 was stabilized by AK301 treatment (Fig 3D). To assess the mechanism of p53 activation, the level of p53 phosphorylation at the ATM/ATR target residue serine-15 was determined [15–17]. We found that AK301 treatment increased phosphorylation at this site (Fig 3D). In addition, ATM was phosphorylated in the presence of AK301 at its autophosphorylation site serine-1981 [18]. Together these data indicate that AK301 activates p53 through a DNA damage response mechanism. To determine if p53-target genes were also being activated, a number of potential target genes were assayed by western blotting (Fig 3E). These data show that Bax, p21 and Mdm2 (but not Bak) were activate by AK301.

### AK301-induced DNA breakage during mitotic arrest

**Fig 4:**
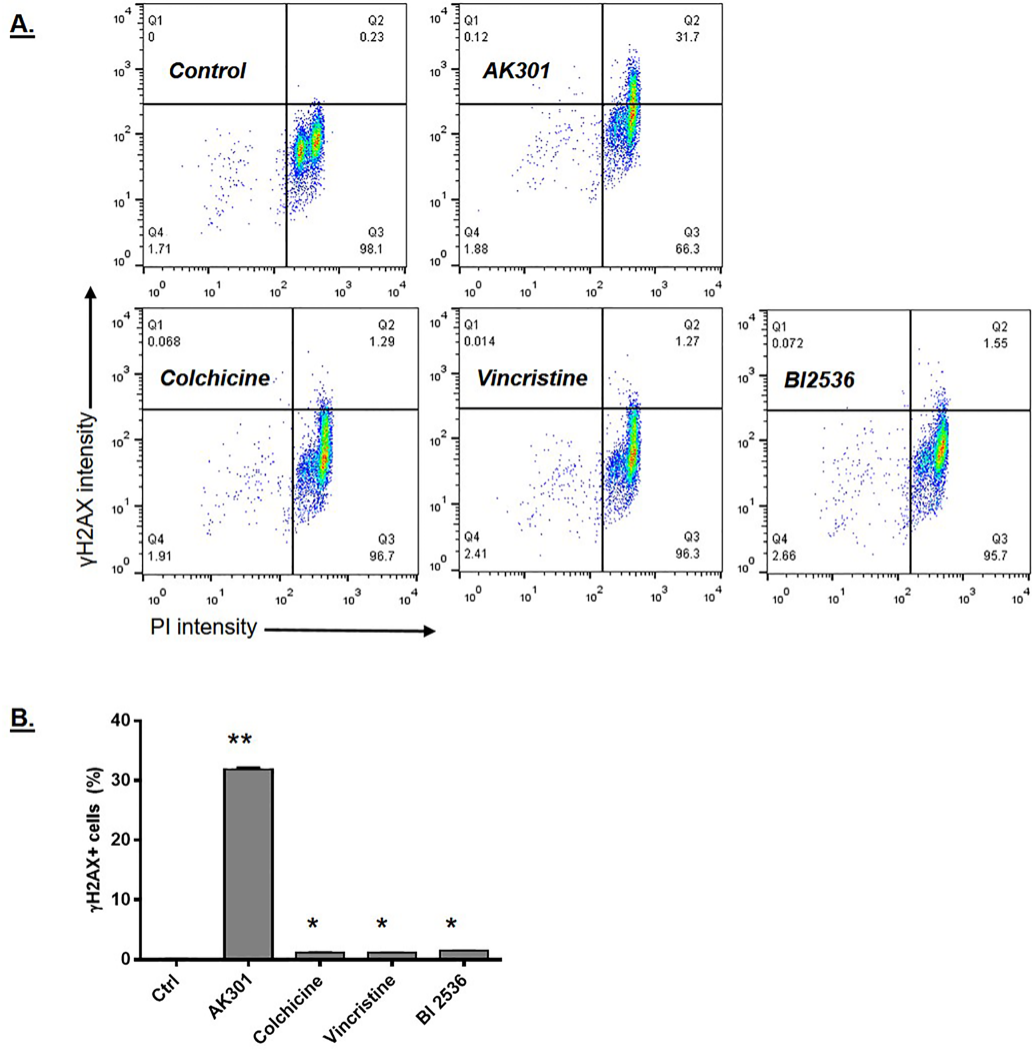
**A)** γH2AX levels in response to treatment with mitotic arrest agents. HCT116 cells were treated for 16 hours with AK301, colchicine, vincristine, or BI2536 at 500 nM. Treated cells were analyzed for γH2AX immunofluorescent staining (Y-axis) and DNA content/PI staining (X-axis) by flow cytometry. **B)** Quantification of γH2AX staining in mitotically arrested cells. Using the gates indicated in 4A, the percentage of cells entering quadrant 2 (Q2) was calculated and compared for the arrest agents shown. AK301-treated HCT116 cells showed a significantly greater proportion of cells with γH2AX activation (*P < 0.0001).

**Fig 5:**
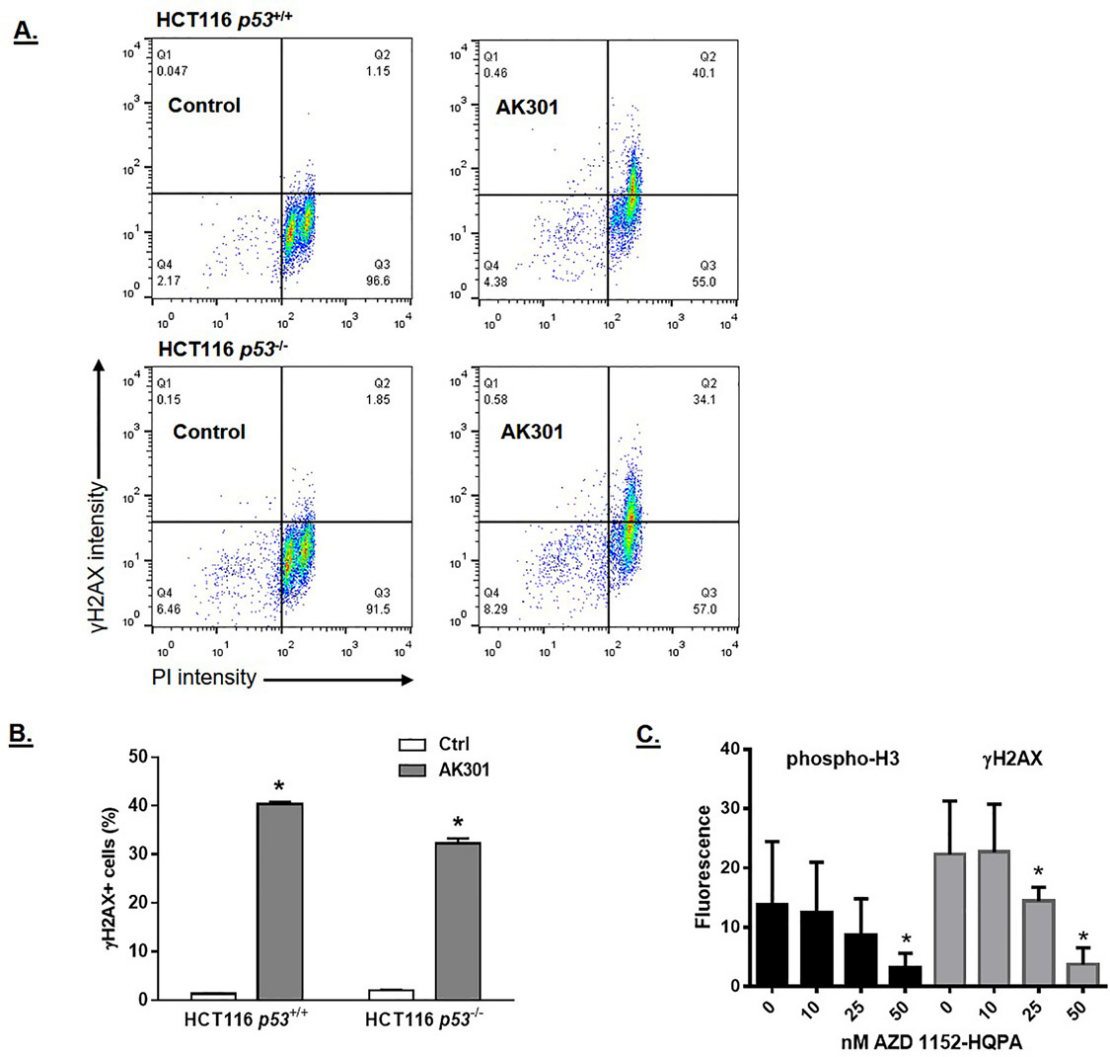
**A)** γH2AX levels in p53-normal and p53-null HCT116 cells treated with AK301. Cells were treated with 500 nM AK301 for 16 hours. Cells were then analyzed for γH2AX immunofluorescent staining (Y-axis) and DNA content/PI staining (X-axis). **B)** Quantification of γH2AX staining in p53-normal and mutant cells mitotically arrested with AK301. Using the gates indicated in 5A, the percentage of cells entering quadrant 2 (Q2) was calculated and compared. AK301-treated HCT116 cells showed a significantly greater proportion of cells with γH2AX activation (*P < 0.0001) but no significant differences between the wild type and null cells. **C)** Aurora B inhibitor-induced reduction in γH2AX. HCT116 cells were arrested with AK301 and then treated with the indicated concentrations of the Aurora B inhibitor AZD1152-HQPA for 1 hour. Immunofluorescent images of γH2AX and phospho-histone H3 staining were then captured and quantified. The Aurora B inhibitor induced a reduction in both phospho-histone H3 Ser 28 (a direct Aurora B target) and γH2AX (*P<0.01).

To further examine the ability of AK301 to induce a DNA damage response, we examined the level of γH2AX [19, 20]. Fig 4A shows a flow cytometry analysis of γH2AX and PI staining of HCT116 cells treated with AK301, colchicine, vincristine or BI 2536. As previously reported, γH2AX staining is higher in G2/M cells than G1 cells [21, 22]. However, γH2AX staining was significantly higher in the AK301 treated cells (32% of AK301-treated cells showed increased γH2AX staining compared to ≈1% with other mitotic inhibitors)(Fig 4B). Analysis of p53-normal and p53-null cells showed a similar level of γH2AX staining both before and after AK301 treatment, which is consistent with the DNA damage occurring prior to p53 activation, and not as a result of p53 (Fig 5A and 5B)[23].

To assess the relationship between mitotic arrest and the DNA damage response, we determined the effect of the Aurora B inhibitor AZD1152HQPA on γH2AX levels [24, 25]. This inhibitor was chosen since it can reduce histone H3 phosphorylation in mitotically arrested cells and promote mitotic chromatin decondensation. As shown in Fig 5C, treatment of cells with AZD1152HQPA decreased histone H3 phosphorylation and γH2AX staining with a similar dose-dependency, consistent with increased γH2AX being linked with the AK301-induced mitotic arrest state. Potential mechanisms that may link mitotic arrest and the DNA damage response are discussed below.

**Fig 6:**
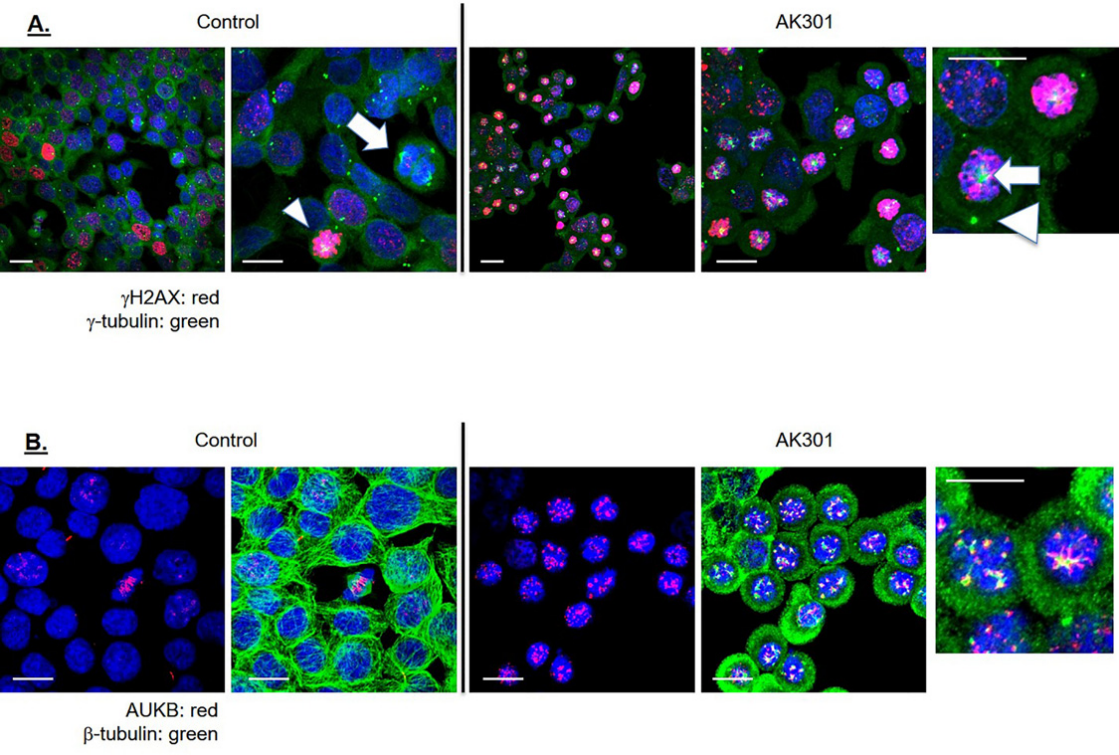
HCT116 cells were examined by immunofluorescence confocal microscopy. Cells were treated with 500 nM AK301 for 16 hours, and then processed for γH2AX and γ-tubulin staining (**A**) or Aurora B and β-tubulin staining (**B**). The color key and 20 μm bars are shown. The arrow and the arrowheads indicate structures referred to in the text. **C**) TUNEL staining shows DNA breakage in AK301-treated cells. HCT116 cells were treated with 500 nM AK301 for 16 hours, and then processed for TUNEL staining. Images of representative field is shown with a 20 μm bar. End-labeled DNA is shown in red and DAPI-stained DNA is blue.

To further confirm the relationship between γH2AX and mitotic arrest, and to define the features of the AK301-induced mitotic arrest state associated with activation of a DNA damage response, AK301 arrested cells were analyzed by immunofluorescent staining and confocal microscopy. Fig 6A shows an immunofluorescent analysis of γH2AX and γ-tubulin in control and AK301 arrested cells. AK301-arrested cells with the highest level of γH2AX staining showed γ-tubulin clustered amongst the condensed mitotic chromosomes. A representative cell showing this feature is indicated by a white arrow in the right-most panel of Fig 6A. A second γ-tubulin foci is also observed in the AK301 arrested cells (arrowhead in Fig 6A). This second foci co-localizes with other centrosome-associated proteins. γH2AX-positive mitotic cells with these features also appear in the control culture, albeit at a much lower frequency (Fig 6A, second panel, white arrowhead). These findings indicate that AK301 arrests cells in a mitotic state that features an active DNA damage response and that cells in this condition occasionally arise in untreated cultures. These data also indicate that arrested cells with the highest level of γH2AX staining have both centrosome-associated and centrosome-independent γ-tubulin foci.

Fig 6B shows Aurora B and microtubule staining in AK301 arrested cells. The arrested cells were found to express elevated levels of chromatin-associated Aurora B. Although the microtubule network in AK301 arrested cells is largely disrupted, short microtubules can be observed in close proximity to the Aurora B foci, suggesting an interaction between the Aurora B/kinetochore complex and microtubules [26]. This finding is consistent with microtubule attachments to mitotic chromosomes in AK301-treated cells. The elevated level of Aurora B expression in AK301-treated cells is consistent with reports showing that this kinase can contribute to ATM activation during mitosis [22].

**Fig 7:**
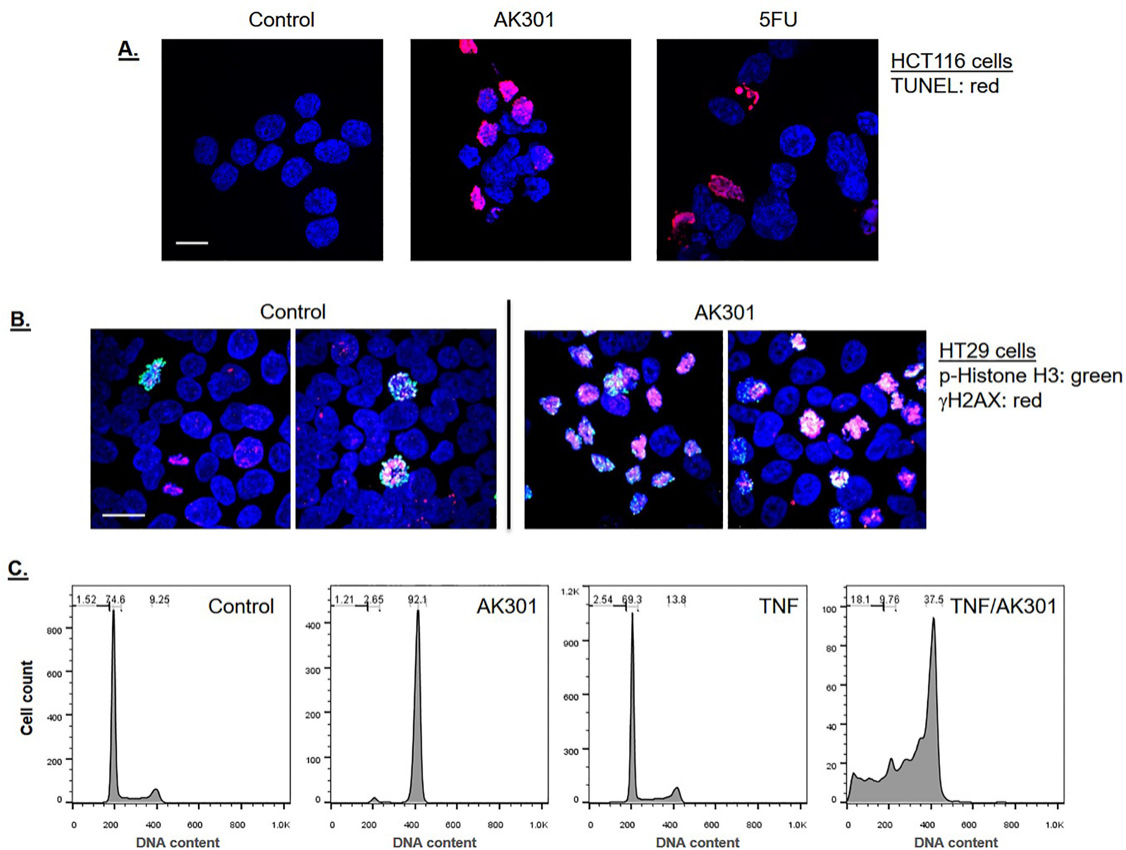
**A)** γH2AX levels in HT29 colon cancer cells following AK301 treatment. HT29 cells were treated with 500 nM AK301 for 16 hours. Treated cells were then analyzed for γH2AX (red) and phospho-histone H3 Ser28 (green) by immunofluorescent staining and confocal microcopy. DAPI-stained DNA is in blue. Two representative images from both control and AK301 treated cultures are shown. A 20 μm bar is shown in the left panel. **B)** Cell cycle analysis of HT29 cells treated with AK301 in the presence or absence of TNF. HT29 cells were treated with AK301 (500 nM) and TNF (50 ng/ml) as indicated for 24 hours. Cells were then fixed and stained with PI for cell cycle analysis by flow cytometry.

Finally, a TUNEL stain was performed to see if the γH2AX staining was associated with detectable strand breakage. As shown in Fig 7A, TUNEL-positive cells are found in the AK301-treated cultures.

### AK301 sensitivity of p53 mutant colon cancer cells

**Fig 8:**
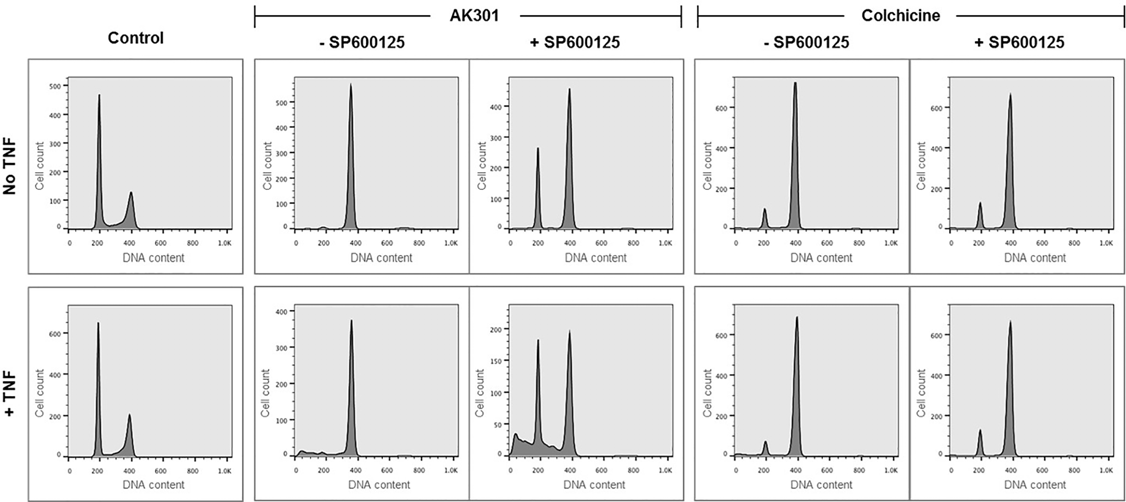
Enhanced TNF sensitivity of AK301-treated HT29 cells after release from mitotic arrest. HT29 cells were arrested in mitosis with AK301 or colchicine. Arresting agents were removed and cells were released from arrest by treatment with the MPS1 inhibitor SP600125 for 2 hours as indicated. Cells were then treated with TNF (as indicated) for 4 additional hours. Under these conditions, AK301-treated cells released from mitotic arrest are the most sensitive to TNF-induced apoptosis as determined by sub-diploid formation.

To examine the generality of the effect of AK301 on colon cancer cells, we tested its effects on the HT29 colon cancer cell line. γH2AX and phospho-histone H3 staining of HT29 cells showed an overlap after AK301 treatment consistent with a DNA damage response in these cells during mitosis (Fig 7B). However, since HT29 cells are p53 mutant, they did not undergo apoptosis following the AK301 treatment-and-release protocol. AK301 did, however, increase the sensitivity of HT29 cells to TNF-induced apoptosis; as shown in Fig 7C, neither TNF nor AK301 alone promoted the formation of sub-diploid apoptotic bodies, whereas a 24 hour co-treatment did (Fig 7C)[8]. Since apoptosis induced by TNF and AK301 is relatively slow (requiring 16–24 hours), and our findings above indicate that cells must exit an AK301 arrest before they undergo apoptosis, we tested whether cells induced to exit mitotic arrest might have a higher TNF sensitivity. Since HT29 cells have a strong mitotic checkpoint and remain in mitotic arrest even after AK301 withdrawal, we utilized the MPS1 inhibitor SP600125 to release them from arrest. As shown in Fig 8, AK301-arrested HT29 cells treated with SP600125 readily exit mitotic arrest [27, 28]. Cells released from mitotic arrest were more sensitive to subsequent TNF treatment than cells that remained arrested (Fig 8). Consistent with our previous results showing a requirement for mitotic release before apoptosis, colchicine arrested cells were neither released from arrest by SP600125 nor were they sensitive to TNF.

### AK301 sensitivity of APC mutant colonocytes

**Fig 9:**
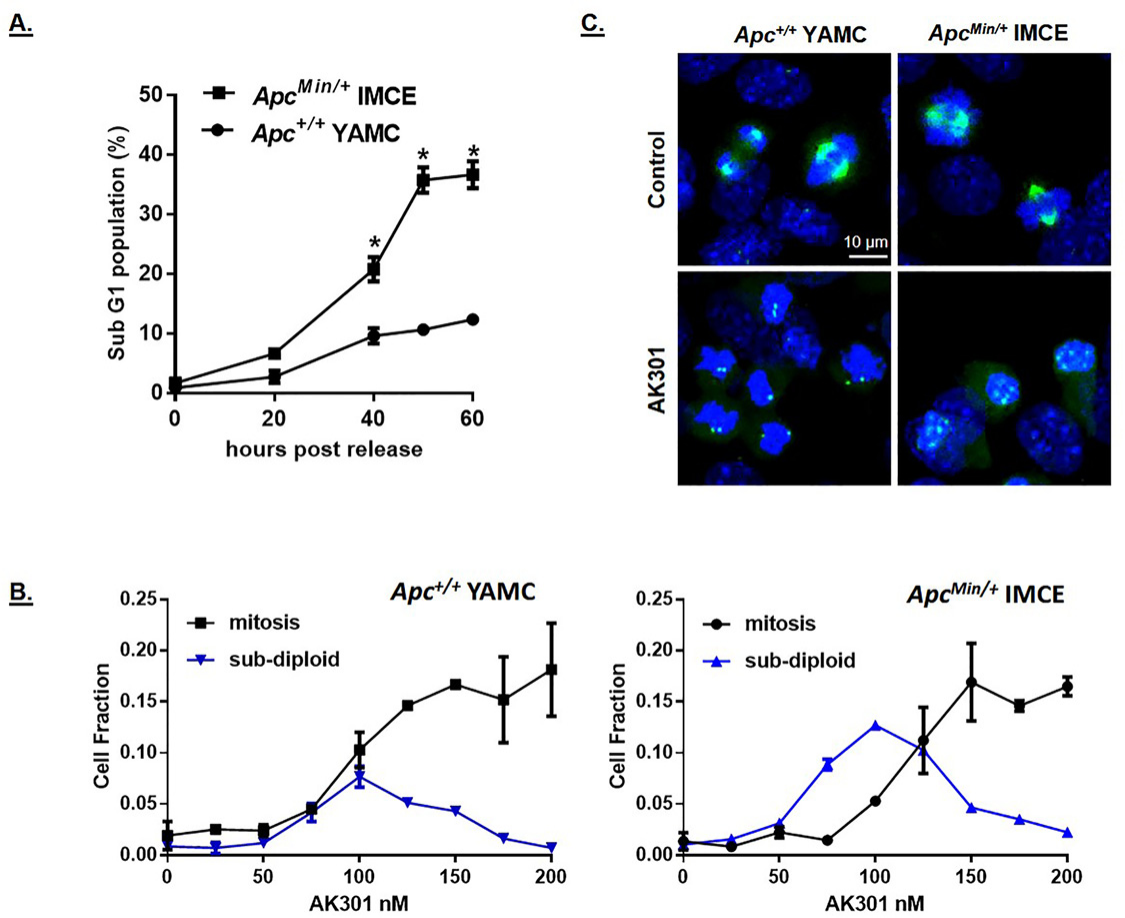
Influence of *APC* mutation on AK301 sensitivity. **A)** Mouse colonocyte cell lines that are *APC*-normal (YAMC) or *APC* heterozygous with a Min mutation (IMCE cells) were treated with 500 nM AK301 for 16 hours. Cells were then released from arrest by medium replacement and analyzed by flow cytometry at the indicated time points. *APC*-mutant IMCE cells underwent apoptosis more readily than wild type cells following release from arrest (*P>0.0001). **B)** Titration of AK301 on *APC*-normal and heterozygous mutant colonocytes. YAMC and IMCE cells were treated with the indicated concentrations of AK301 for 16 hours and then assessed for sub-diploid formation by flow cytometry and for mitotic arrest by the phospho-histone H3 staining. Significantly higher levels of apoptosis were observed for IMCE cells at AK301 concentrations from 75–125 nM. **C)** Comparison of Aurora A localization in YAMC and IMCE cells treated with 100 nM AK301. Cells were immunostained for Aurora A (green) with nuclei counterstained with DAPI (blue). Untreated YAMCs and IMCE cells show a normal bipolar localization of Aurora A to the centrosome and spindle in mitotic cells. AK301 treatment of YAMCs restricted Aurora A association with the centrosome, whereas treatment of IMCE cells induced the formation of multiple diffuse Aurora A foci.

Since the APC protein is involved in microtubule elongation and mitotic spindle assembly and AK301 targets these processes, we tested the sensitivity of *APC*-normal and *APC*-mutant mouse colonocyte cell lines to AK301 (YAMCs and IMCEs, respectively)[10, 11]. Release from AK301 arrest by compound withdrawal resulted in a significantly higher level of apoptosis in *APC*-mutant IMCE cells compared to *APC*-normal YAMCs (Fig 9A). Interestingly, titration of AK301 on these two cell lines showed that apoptosis occurred at compound concentrations lower than those required to induce optimal mitotic arrest, and cell death could occur without compound removal (Fig 9B). Moreover, this sensitivity was more pronounced in the *APC*-mutant cell line. Since apoptosis at the lower AK301 concentrations may result from a disruption in mitotic progression, we analyzed the structural features of *APC*-normal and mutant cells to AK301. We tested a number of antibodies and found that staining for total Aurora A, which associates with the centrosome and mitotic spindle, showed a high degree of disruption in *APC*-mutant cells. In untreated cells, Aurora A interacted with the centrosome and the mitotic spindle, regardless of *APC* status (Fig 9C top panels)[29]. However, following AK301 treatment, Aurora A interaction was discreetly localized to centrosomes in *APC*-normal cells, but dispersed into multiple, disorganized foci in the *APC* mutant cells (Fig 9C, bottom panels). These data suggest that a more severe mitotic disruption in *APC*-mutant cells underlies their increased sensitivity.

## Discussion

We previously reported the identification of a family of small molecules that induce mitotic arrest in colon cancer cells and increase their sensitivity to TNF and other death ligands [8, 9]. In this study, we show that the most potent of these compounds, AK301, is also effective at inducing a mitosis-to-apoptosis transition in the absence of a death ligand in p53-normal colon cancer cells. In this instance, apoptosis can be induced by treating cells with AK301 to induce arrest, and then withdrawing the compound to release cells from arrest and into apoptosis. AK301 appears to function by arresting cells in a mitotic state in which a DNA damage response is activated and p53 is stabilized. Compound withdrawal then allows progression to apoptosis, which likely entails the activation of p53-target genes following the decondensation of mitotic chromatin [30]. Although cells arrested in mitosis by other agents have been reported to activate ATM and components of the DNA damage response pathway, AK301 arrests cells in a state in which this response is especially robust [21]. In addition, mitotic arrest by AK301 is readily reversible, which facilitates the transition to apoptosis following AK301 withdrawal. The apoptotic signaling pathway activated by AK301 may be exploitable for cancer treatment, particularly for cancer cells with defects in the mitotic apparatus and mitotic checkpoints.

To better understand the relationship between mitotic arrest and the DNA damage response, we analyzed the arrest state generated by AK301. AK301-arrested cells with high levels of γH2AX displayed condensed chromatin adjacent to a central cluster of γ-tubulin. However, this γ-tubulin was not centrosome-associated. Instead, the centrosomes (and their associated γ-tubulin) remained at the cell periphery with minimal migration to the mitotic poles. Cells in this state are common in AK301 cultures but also appear in untreated cultures, albeit at much lower frequency, indicating that cells encounter this type of arrest during a normal cell division. How the DNA is broken under these conditions is not clear. One possibility is that γ-tubulin-seeds the formation of aberrant spindles that pull the chromosomes to induce breakage. Consistent with this possibility, we find microtubules in close association with the Aurora B passenger protein in AK301-arrested cells, indicating that microtubule attachment to the kinetochore is being established [26]. The relationship between microtubule attachment and γH2AX staining is also supported by the finding that colchicine-treated cells have completely disassembled microtubules and lower levels of γH2AX. Although microtubule pulling is one possible mechanism for ATM activation, Aurora B can also directly activate ATM in the absence of DNA breakage [22]. Since AK301 arrested cells show elevated levels of Aurora B, it is possible that direct activation of ATM by chronic Aurora B activity may be occurring, thereby accentuating the DNA damage response. Our finding that the Aurora B inhibitor can reduce γH2AX levels in AK301-treated cells is consistent with this possibility. Understanding how the DNA damage response is optimally activated during mitotic arrest could provide insight into how this event might be best targeted to cancer cells with mitotic defects.

AK301 was originally identified by virtue of its ability to arrest cells in a mitotic state that is highly sensitive to the apoptotic actions of TNF [8, 9]. The basis of this sensitivity was determined to be an increased coupling between TNFR1 and capase-8 activation [8]. Based on our findings here, and reports by other groups, the increased caspase-8 activation by TNF in the presence of AK301 may be mediated in part by ATM activation; siRNA knock down of ATM has been reported to reduce TNF-induced caspase-8 activation in HeLa cells [31]. The synergistic action of the TNF and AK301 may also result in part from the ability of TNF to promote cells to exit mitotic arrest and enter apoptosis. Caspase activation by death ligands has been reported to promote degradation of the spindle checkpoint proteins, which releases cells from mitotic arrest and allows them to enter apoptosis [32]. We find that cells arrested by AK301 must first exit mitosis before they can enter apoptosis and TNF may facilitate this exit from mitotic arrest.

Our findings here suggest that the impact of AK301 on cells is complex and depends on a range of genetic and tissue-specific factors. For instance, cells with a normal p53 gene but a weak spindle assembly checkpoint may be more directly sensitive to AK301. In addition, our analysis indicates that cells carrying an *APC* mutation are more sensitive to AK301, compared to p53-mutant cells with a robust SAC that may require a death ligand to undergo apoptosis. *APC* mutations frequently occur at an early stage of colon cancer development and are well documented to increase Wnt signaling by increasing β-catenin stability [33–35]. However, the APC protein is also known to facilitate spindle assembly during mitosis by stabilizing microtubule plus (growth) ends [36–40]. C-terminal truncating mutations of *APC*, most commonly found in colon cancers, act in a dominant negative fashion to disrupt spindle assembly [41, 42]. Since AK301 also affects microtubules, we tested the sensitivity of *APC*-normal and mutant colonocytes to AK301 and found that the mutant cells were more sensitive. The basis of this higher sensitivity is not entirely clear, but may result from a more severe disruption of mitotic events resulting from the dual targeting of the mitotic spindle. Consistent with this possibility, we observed a more severe disruption of centrosome regulation and localization (using total Aurora A staining) in *APC*-mutant cells treated with AK301. How this disruption is translated into increased apoptosis in these and other *APC*-mutant cells is presently under study.

Although microtubule and mitosis targeting chemotherapies are not typically used to treat colon cancer, our findings here, and reports by other groups, suggest that these therapies may in some cases be advantageous [43–45]. Colon cancers with microsatellite instability are usually p53-normal and have a defective CHFR mitotic checkpoint, and are therefore an interesting target for AK301 (and similarly acting agents), particularly if they also have an *APC* mutation. Although colon cancers with this combination of defects represent only a subset of all colon cancers, the specific targeting of this type of cancer may provide an avenue for patient stratification. Moreover, microsatellite unstable colon cancers are particularly interesting since they are poorly responsive to present 5-fluorouracil-based therapies [46, 47]. Understanding how AK301 targets cellular components to induce a reversible mitotic arrest state that includes elevated levels of ATM signaling and p53 stabilization could provide valuable information into how cancers with the appropriate vulnerability might best be targeted.

## Abbreviations

ATM: ataxia telangiectasia mutated
IMCE: Immorto-Mouse Colon Epithelial cells
MTOC: microtubule organizing center
pHH3: phospho-histone H3
PI: propidium iodide
SAC: spindle assembly checkpoint
TNF: tumor necrosis factor
TNFR1: TNF receptor 1
YAMC: Young Adult Mouse Coloncytes
